# Consciousness: Matter or EMF?

**DOI:** 10.3389/fnhum.2022.1024934

**Published:** 2023-01-18

**Authors:** Johnjoe McFadden

**Affiliations:** Faculty of Health and Medical Sciences, University of Surrey, Guildford, United Kingdom

**Keywords:** consciousness, neuroscience, EEG, electromagnetic, cognition, theory

## Abstract

Conventional theories of consciousness (ToCs) that assume that the substrate of consciousness is the brain's neuronal matter fail to account for fundamental features of consciousness, such as the binding problem. Field ToC's propose that the substrate of consciousness is the brain's best accounted by some kind of field in the brain. Electromagnetic (EM) ToCs propose that the conscious field is the brain's well-known EM field. EM-ToCs were first proposed only around 20 years ago primarily to account for the experimental discovery that synchronous neuronal firing was the strongest neural correlate of consciousness (NCC). Although EM-ToCs are gaining increasing support, they remain controversial and are often ignored by neurobiologists and philosophers and passed over in most published reviews of consciousness. In this review I examine EM-ToCs against established criteria for distinguishing between ToCs and demonstrate that they outperform all conventional ToCs and provide novel insights into the nature of consciousness as well as a feasible route toward building artificial consciousnesses.

“*I'm not asking you, I'm telling you. These creatures are the only sentient race in the sector and they're made out of meat*.”

Terry Bison “They're made out of meat” (Bisson, 1995).

## Introduction

In their recent article “Hard criteria for empirical theories of consciousness.”

Doerig et al. (2021) argue that there are now a wealth of theories of consciousness (ToCs) but no established stringent criteria by which each could be compared. They proposed a list of criteria through which ToCs could be checked and compared. They are that any TOC should:

1. Address paradigm cases of consciousness, such as optical and auditory illusions or masking, when the same sensory information can switch between being either conscious or non-conscious.
2. Cope with the “folding argument” which applies to ToCs that associate consciousness with some kind of information processing architecture in the brain, such as recurrent thalamocortical interactions (Nervous et al., 1998; Lamme and Roelfsema, 2000). The folding argument points out that both recurrent and feedforward networks can approximate any input-output mathematical function (Doerig et al., 2019), so a recurrent network could be “unfolded” into a feedforward network without any change in inputs or outputs.
3. Cope with the small network argument which arises from the observation that many ToCs imply that small networks with fewer than ten neurons are conscious. The authors admit that many ToCs argue that small networks lack additional key ingredients, such as complexity or size of the network. However, these are arbitrary “curve-fitting” additions to the theory, rather than being predicted by the theory, that add complexity to the ToC. I additionally point out that Bayesian inference, which is today considered to be fundamental to scientific reasoning (Howson and Urbach, 2006; McFadden, 2021a,b), automatically incorporates a preference for simple solutions, not as priors, but as part of the likelihood function that delivers higher posterior probabilities to simpler theories or models that fit the data but make sharper predictions than more complex theories.
4. Copes with the multiple realization argument (Bechtel and Mundale, 1999) which deals with the problem of understanding why some complex systems, such as human brains, are consciousness, whereas other complex systems, such as robots or mammalian immune systems, are presumed to be non-conscious. The challenge is whether a ToC makes clear-cut and specific predictions about which systems, other than human brains, are conscious.

Doerig, Schurger and Herzog do not claim that their criteria are exhaustive but only that they provide “a first set of guidelines to foster discussions about consciousness as an empirical phenomenon.” In a more recent review, Seth and Bayne (2022) independently suggest several additional criteria, some of which overlap with the S&B criteria but others are distinct and are added here. Additionally, I add two additional criteria (^*^'ed) to make eleven tests of ToCs.

5. Addresses the unity of consciousness (Seth and Bayne, 2022) often known as binding problem (Hardcastle, 1994; Singer, 2001; Seth and Bayne, 2022) of how “the experiences that a single agent has at a time seem always to occur as the components of a single complex experience, one that fully captures what it is like to be that agent.” This is particularly puzzling because we know that the information in the conscious mind at any single moment is encoded in widely-separated neurons in different regions of the brain. Yet integration of information is intrinsic to conscious perception such that, or example, it is impossible to consciously conceive of an object that lacks color. This would present no problem to a computer, nor does it present any problem to the non-conscious mind that, for example, reacts to objects approaching our eye by blinking, irrespective of the object's color. Yet, in the conscious mind, color is intrinsically bound up with visual objects such that, in synaesthesia, even sounds have colors. The binding problem is then that of understanding how diverse information encoded by firing rates of widely distributed neurons is bound into a singular unified conscious experience. Note that the need to address the binding problem is implicit in many ToCs, though not always stated. For example, theories that invoke integrated information as a key feature of consciousness, such as Integrated Information Theory, IIT (Tononi, 2004) implicitly assume that complex conscious information is bound into a single integrated conscious state.
6. Addresses neural data such as the apparent absence of conscious experience in certain regions of the brain, such as the cerebellum or during certain states of the brain, such as grand mal or absence epileptic seizures (Seth and Bayne, 2022). I include here also timing data, such as the psychological refractory period for conscious awareness or the attentional blink or postdictive effects on conscious perception. Several of these neurological phenomena can be grouped into the broader problem of why neural correlates of consciousness (NCCs), are NCCs.
7. Addresses the measurement problem (Seth and Bayne, 2022) of identifying trustworthy measures of consciousness that could, for example, be used to measure the degree of consciousness in a patient or in artificial systems, such as cerebral orgenanoids or AI.
8. ^*^Accounts for how and why a single brain operates in two modes: a non-conscious parallel processor and a conscious serial processor. As Baars (1993) put it how does “a serial, integrated and very limited stream of consciousness emerge from a nervous system that is mostly unconscious, distributed, parallel and of enormous capacity.” Baars pointed out that our non-conscious mind is capable of a massive degree of multitasking, such as directing the delicate limb muscle movements needed to ride a bicycle whilst simultaneously calculating and coordinating the precise movements of the lips, tongue and nasopharynx needed to sing a familiar tune. Yet, our conscious mind can only do one thing at a time. It is, for example, not possible to chat to a friend whilst simultaneously performing long division in your head, a challenge that would be trivial for any chatbot. Any ToC must account for how, and why, these very different modes are generated from the same neural substrate in the brain. I also include here the related curious feature, often overlooked by ToC's, that consciousness appears to be required for learning novel skills, such as riding a bike, but, once leant, those skills can operate without conscious control (McFadden, 2006).
9. ^*^Distinguishing intelligence from consciousness. This criterion was first put forward by Block (2009) who argued that most ToCs fail to distinguish between the intelligence delivered by diverse complex systems including computers, the non-conscious mind, AI, and consciousness.
10. Accounts for the emergence of consciousness through natural selection (Seth and Bayne, 2022).
11. Whether the ToC is able to make novel testable predictions (Seth and Bayne, 2022).

First, a brief introduction to EM field theories of consciousness. The idea that the conscious mind is some kind of field goes back at least as far as the early twentieth century gestalt psychologists who emphasized the holistic nature of perception, or *gestalts*, and argued that they must be encoded in some kind of field, rather than discontinuous particles (McFadden, 2013a). The idea was further developed and extended by Popper et al. (1993) who proposed that consciousness was a manifestation of some kind of overarching *force field* in the brain; whilst the neurobiologists Libet (1994, 1996) and (Lindahl and Arhem, 1994) called it the “conscious mental field.” Although each of these authors accepted that consciousness must be some kind of field in the brain they nevertheless concluded that it could not be any of the known physical fields, so its nature remained mysterious.

Most neurobiologists saw this as a unwelcome return to Cartesian dualism and opted instead for the monist position that both mind and consciousness are seated in the matter of the brain. That the brain also generates an EM field had been known from the late 19^th^ century but it was, and still is, generally assumed to play no more role in brain function that that of a steam whistle on the operation of a steam engine. Nevertheless, because of its accessibility, particularly after the invention of electroencephalography (EEG) and magnetoencephalography (MEG), the brain's EM field was (and is) adopted as a routine measure of the level consciousness in, for example, anesthesia (Roth, 1951) and comatose patients (Loeb, 1958).

In his 1994 book “The Astonishing Hypothesis” the Nobel laureate and co-discoverer of the double-helical structure of DNA, Crick (1994) argued that science was capable of tackling the problem of consciousness and proposed starting with an initial focus on identifying neural correlates of consciousness (NCCs). This programme was enthusiastically adopted by a new generation of neurobiologists who searched for NCCs amongst anatomical sites, patterns of neural firing or architecture of neural processing using EEG as well as more advanced techniques such as functional magnetic resonance imaging (fMRI) and magnetoencephalography (MEG) together with intracellular and extracellular recordings. One of the most surprising results to emerge from these studies was that attention and awareness tended to be associated, not with neural firing *per se* or the anatomical site of neural firing, but with the synchronicity of firing of multiple neurons (Gray et al., 1989; Crick and Koch, 1992; Crick, 1994). For example, work conducted by Wolf Singer and colleagues demonstrated that neurones processing visual information fired asynchronously when an animal does not attend to the stimulus, but fired synchronously when the animal attends to, and is presumed to be conscious of, the stimulus (Kreiter and Singer, 1996). Numerous subsequent studies, recently summarized (McFadden, 2020), have confirmed and extended these findings so that, even today, synchronous neural firing and the EM fields that it generates remains the best NCCs. The questions is, why?

In 2000, both McFadden (2000) and Pockett (2000) published books which proposed a possible solution. They pointed out that if a large group of neurons is firing asynchronously then their net EM field will be subject to destructive interference and sum to zero: very little information from these neurons will be *transmitted* to the brain's EM field. If those same neurons are firing synchronously then the net EM field generated by their activities will be subject to constructive interference, so the information they encode will be effectively transmitted into the brain's EM field. The combination of destructive and constructive interference thereby provides a synchronicity filter that ensures that the brain's EM field is dominated by information encoded in synchronously-firing neurons. So, whereas the matter of the brain encodes both conscious and non-conscious neuronal information, its EM field will be dominated by the much smaller stream of information encoded by synchronously-firing neurons—precisely those neurons that were identified as prime NCCs. It is a small step from this realization to the proposal that the seat of consciousness is not the matter of the brain but the equally physical yet immaterial, brain EM field generated by synchronous neuronal firing.

Both McFadden J. (2002), McFadden J. J. (2002) and Pockett (2002), elaborated on this idea in papers published in 2002 pointing out that electromagnetic field ToCs (EMF-ToCs to contrast with neural-ToCs that propose that consciousness is encoded in the matter of neurons) easily solve the unity or binding problem (criterion 8 above) since EM fields automatically integrate their encoded information into a single physical field. Around the same time, similar theories were proposed by the neurophysiologist John (2001, 2002) and the neurophysiologists (Fingelkurts et al., 2001, 2013; Fingelkurts and Fingelkurts, 2008). In the following years, several other EMF-ToCs have been published (Barrett, 2014; Jones, 2016, 2017; Liboff, 2016; Zhakenovich et al., 2016; Hales, 2017; Hunt and Schooler, 2019; Keppler, 2021; Detmar, 2022). Despite this, none of the ToCs discussed in either the Doerig et al. (2021) paper or the Seth and Bayne (2022) review are EMF-ToCs, a deficiency that is rectified here.

In this paper, I review the principal EM-ToCs against the criteria proposed by both Doerig et al. (2021) or Seth and Bayne (2022). Broadly, EMF-ToCs are defined as those ToCs that propose that the seat of consciousness resides in the brain's EM field, rather than its neuronal substrate. To identify EMF ToCs I performed a Google Scholar search with the terms “electromagnetic field theory” +consciousness for the period 2010—present. This returned 336 hits. From these I identified nine EMF-ToCs (John, 2002; McFadden J., 2002; McFadden, 2020; Pockett, 2002, 2012; Fingelkurts et al., 2013; Barrett, 2014; Zhakenovich et al., 2016; Hales, 2017; Hunt and Schooler, 2019; Keppler, 2021) that are discussed here. This is not intended to be an exhaustive review of the EM-ToC literature but an examination of how well they, as a group, fare against recently-established criteria for evaluation of ToCs. A key dividing line within EMF-ToCs is those that predict that conscious brain EM fields influence behavior (John, 2002; McFadden J., 2002; McFadden, 2020; Fingelkurts et al., 2013; Barrett, 2014; Zhakenovich et al., 2016; Hales, 2017; Hunt and Schooler, 2019; Keppler, 2021) which I term type 1 or EMF_1_-ToCs^1^, and those, such as Pockett's theory (Pockett, 2011, 2012), which predict that conscious brain EM fields do not influence behavior, which I term type 0 or EMF_0_-ToCs. A third category are those EMF-ToCs that are agnostic on the question of whether consciousness influences behavior, which I term EMF-ToC_x_ theories. EMF-ToC_x_ may agree with the predictions of EMF-ToC_1_s and EMF-ToC_0_s so will not be dealt with separately. I contrast EMF-ToCs with neural-ToCs and discuss how EMF-ToCs address both Doerig, Schurger and Herzog's, Seth and Bayne's ToC test criteria plus a few additional criteria proposed here.

## Results

1. Both type one and type zero EMF-ToCs address paradigm cases of consciousness. As already pointed out, synchronous neuronal firing, which is the primary source of the brain's EM field, strongly correlates with conscious perception. However, the most accessible measure of the brain's EM field is *via* EEG or MEG signals which are generated by synchronous neuronal firing. The strong correlation between EEG signals and conscious states is evidenced by the widespread clinical use of EEG to assess the level of consciousness and awareness in brain-damaged patients (Engemann et al., 2018) and in general anesthesia (Musialowicz and Lahtinen, 2014). EEG signals—and thereby the brain's EM field—also correlate with perception in change blindness (Koivisto and Revonsuo, 2003), perceptual masking (Schubert et al., 2009), sound-induced flash illusions (Kaiser et al., 2019) and classic perceptual switching such as when viewing the Rubin's vase/face illusion (Müller et al., 2000). EEG alpha wave perturbations are also correlated with “mind wandering” (Compton et al., 2019) when attention drifts away from a task activity. The consistent correlation between EEG/MEG and conscious acts of volition is currently being harnessed to construct prosthetic devices that are controlled by a patient's EEG (Al-Quraishi et al., 2018). All of these and many more EEG studies provide strong evidence that the brain's EM field remains the most reliable correlate of consciousness, consistent with all EMF-ToCs.

Note that the tight correlation of EEG signals to perception and conscious states is not accounted for by a simple correlation of EEG to activation of particular neural pathways or ensembles that are conscious for reasons unrelated to the EM fields they generate, such as that they have the highest value of phi as described by Integrated Information Theory (IIT) (Tononi, 2004) or involve critical thalamocortical recurrent loops (Diseases et al., 1998). This is due to the inverse problem of being unable to predict the pattern of neural firing responsible for generating a particular EEG signal, solely from the EEG signal (Grech et al., 2008; Baillet, 2014). This follows from the physical principle that potential infinite combinations of electrical sources can generate the same EMF (Jackson, 1999). So, if a particular EEG signal was a correlate of consciousness solely because it happened to generated by a neural firing pattern that, in itself, conferred consciousness (the brain EMF that gave rise to the EEG signal was neither sufficient nor necessary for consciousness), that correlation would likely be diluted by diverse non-conscious neural firing patterns that just happen to generate the same EEG signal. Conversely, if a putative neural firing pattern is a sufficient and necessary cause of consciousness, it is unlikely to consistently generate a EEG correlate of consciousness due to destructive interference from adjacent neural firing networks. Consistently high levels of correlation found between EEG signals and conscious states is only guaranteed if the state of the brain's EM field, rather than the state of the neurons that generate the brain EM fields, is both necessary and sufficient for consciousness.

This conclusion is consistent with recent remarkable findings by Pinotsis and Miller (2022) that demonstrate that, although the exact neurons (the neural ensemble) maintaining a given memory in working memory varies from trial to trial, what is known as representational drift, stability of working memory emerges at the level of the brain's electric fields as detected by EEG. Since working memory is considered to be, essentially, conscious memory, all EMF-ToCs predict that it resides in the brain's EM fields rather than in its neurons, acting as the brain's global workspace (Baars, 2005), consistent with Pinotsis and Miller's findings. The higher level of correlation between the contents of working memory and the brain's EM fields, rather than the state of the brain's matter-based neurons, is a considerable challenge to all neural-ToCs.

2. EMF-ToCs are impervious to the unfolding argument since they are dependent on neither feedforward nor recurrent pathways. Indeed, due the uncoupling between brain neural activity and brain EM fields discussed above (resulting from the inverse problem), its seems likely that identical EM fields, and thereby the same conscious state, could be generated by feedforward or recurrent networks.
3. EMF_1_-ToCs, such as the cemi field theory, cope with the small network argument by predicting that small networks are non-conscious. This follows from the theory's insistence that, to be reportably conscious, brain EM field-based information must, either directly or indirectly, influence the firing of motor neurons (McFadden J. J., 2002). There are sound theoretical grounds (McFadden J., 2002) and abundant experimental evidence that neural firing rates are indeed influenced by the brain's endogenous EM field [summarized in my recent paper (McFadden, 2020)]. In the cemi field theory, that influence is proposed to be experienced as what we call our “free will,” the output of our conscious mind (McFadden, 2021c). So, in EMF_1_-ToCs, neurons act as both transmitters and receivers of EM field-based conscious thoughts forming the strange loop proposed by philosopher Hofstadter (1979, 2007) to be central to consciousness.

Field gradients of 2–4 mV/mm appear to be necessary to influence neural firing (Frohlich and McCormick, 2010) which is similar to the strength of the endogenous brain EM fields (McFadden J., 2002) that can be detected by EEG. Thousands or millions of aligned neurons must fire synchronously (McFadden J., 2002; Pockett, 2002) to generate fields of as strong as 2–4 mV/mm. Small networks will generate only very weak field gradients that will usually be insufficient to influence neural firing patterns and will thereby be non-conscious.

It is less clear that type 0, or EMF_0_-ToCs, for example, Pockett's (2000, 2002) EM-ToC are resistant to the small network argument. As Pockett (2002) has argued, from the perspective of EEG, small networks will be drowned out by the field generated by large coherent networks but, without a reportability threshold, within EMF_0_-ToCs I see no reason to exclude consciousness from the experience of small networks just because they generate field gradients below the sensitivity of EEG.

4. Just as neural-ToCs that locate consciousness in the matter of the brain are susceptible to the multiple realization argument (MRA), since not all matter is conscious, EMF_0_-ToCs are similarly subject to the MRA since not all EM fields are likely to be conscious. EMF_0_-TOCs generally resort to the same kind of primary defense against rampant panpsychism as neural-ToCs by insisting that some additional criterion, such as complexity, integration, anatomical location, informational processing architecture or access to working memory, is needed for consciousness. Since thoughts are informationally-rich, the complexity criterion is indeed sound: what could a single bit encoded by a single particle of matter, or EM field sine wave, possibly *think*? The substrate of consciousness must, at a minimum, possess sufficient complexity to encode a thought. However, neither neural-ToCs nor EMF_0_-ToCs provide objective criteria for determining the level of neuronal complexity, or any other criterion, needed for neural computation to reach conscious, except, as in IIT (Tononi and Koch, 2015), to propose it is the winner of some kind of internal neuronal mathematics competition.

In contrast, EMF_1_-ToCs provide an objective criterion for distinguishing conscious from non-conscious EM fields. This arises from the requirement that, to be reportably conscious, a system must be able to generate (rather than merely transmit) thoughts as gestalt (integrated) information (McFadden, 2013b)—our thoughts—that can be communicated to the outside world *via* a motor system. This simple formula excludes consciousness from artifacts such as toasters, computers and other AI devices that, although capable of generating complex EM fields are designed to avoid electromagnetic interference (EMI) with their operation. A strong prediction of EMF-ToCs is therefore that conventional computers that exclude EM field influences on their outputs will never be conscious. If demonstrably conscious conventional computers are ever developed, then EMF_1_-ToCs will immediately be falsified. Type 1 EMF-ToCs also propose an objective means of assessing the level of consciousness (the degree by which actions are controlled by the conscious, rather than the non-conscious, mind) in different brains by measuring the correlation between a nervous system's motor outputs and its brain's EM fields. The strong correlation between willed actions and EEG signals in humans has already been known since Libet et al. (1982, 1983a,b) pioneering experiments in the 1980's that demonstrated that EEG signals can be used to predict intentions to act, prior to the subject knowing their intention, findings that have inspired recent research efforts to build EEG-operated brain-computer interfaces (BCIs) for patients with prosthetic limbs (Guger et al., 1999) or suffering from locked-in syndrome (Aricò et al., 2018). Unfortunately, only limited studies of the correlation between EEG and behavior have been performed in primates (Attaheri et al., 2015), cats (Engemann et al., 2018), horses (de Camp et al., 2020), and a scattering of other animals (Klemm, 1992); but, type 1 EMF-ToCs do, at least, provide a framework through which the question of the level of consciousness in animals could potentially be measured.

5. McFadden recently argued that binding is a property not only of visual information but all sensory information (McFadden, 2020) and is fundamental to the gestalt information processing (McFadden, 2013b) of ideas and concepts that is characteristic of the conscious mind and contrasts with the digital processing capabilities of non-conscious minds. The binding problem is then that of understanding “*our capacity to integrate information across time, space, attributes, and ideas*” (Treisman, 1999). Nearly all neuronal ToCs argue that binding, or integration, of information in the conscious mind is a consequence of some forms of neural processing, for example, synchrony (Engel et al., 1999) or hypersynchrony (Mashour, 2004) of neural firing, the formation of neuronal assemblies through processing the same sensory information, or the involvement of particular information processing architectures such as reentrant loops (Singer, 2001), or involving particularly parts of the brain, such as the thalamus (Hardcastle, 1994) or is accomplished through some hypothetical structure, such as the global workspace (Baars, 2005) or, like IIT (Tononi, 2004), claims that consciousness is associated with particular mathematical properties of information processing in the brain. Yet, as the physicist Rolf Landauer argued “information is physical.” Integrated information must therefore be physically integrated. Matter encoded information is always discrete and digital in nature, except in exotic quantum mechanical states, such as a Bose-Einstein condensate, which are completely infeasible in the brain (McCrone, 2003). No neural-ToC provides an adequate account of how information encoded in discrete matter is integrated in the conscious mind. Classically-encoded information is only physically integrated in the energy fields generated by matter (McFadden, 2020). For example, the gravitational field that keeps our feet on the ground represents an integration of the mass of our body and that of the entire planet, despite the fact that nearly all the atoms and molecules of matter that form that field is located thousands of miles from the soles of our feet. EM-ToCs pointed out, more than 20 years ago (McFadden J., 2002; McFadden J. J., 2002), and without recourse to special states of matter, hypothetical structures or complicated mathematical functions, that the brain's EM field automatically integrates neural information into a singular non-material physical field thereby effortlessly solving the binding problem.
6. Addresses neural data such as the apparent absence of consciousness in the cerebellum and providing an explanation of why NCCs, such as neural synchrony, correlate with conscious awareness. I have discussed above how the recognition that information encoded in synchronous neural firing will dominate the brain's EM field was the primary inspiration for a renewal of interest in EMF-ToCs in the first decade of the 21^st^ century. EMF-ToCs provide the most parsimonious explanation of why neural synchrony is a NCC and are thereby favored by Occam's razor (McFadden, 2021d) since, unlike neural ToCs, they predict, rather than merely incorporate, a strong association between synchronous neural firing and consciousness. EMF-ToCs also account for why neural activity in the cerebellum appears to be non-conscious. This is likely due to the cerebellum's intricate folding, compared to cerebral cortex, which ensures that currents arising in neighboring patches of cerebellum activation tend to be running in opposite directions resulting in cancellation of their EM fields through destructive interference. The same reason is thought to be responsible for the invisibility of the cerebellum in EEG or MEG measurements (Andersen et al., 2020).

EMF-ToCs also account for the lack of consciousness in absence epileptic seizures in which patients lose consciousness. These are associated with strong regular and usually bilaterally synchronous and symmetric EEG signals particularly in the 2–4 Hz range (Hedström and Olsson, 1991). Naively, one might expect that that EMF-ToCs might predict that strong EEG signals would be associated a heightened, rather than reduced state of consciousness. However, in contrast to the information-rich EM-encoded information detectable in a normal EEG, which correlates with sensory information, perception and the contents of consciousness, the highly rhythmic EMF fluctuations characteristic of EEG seizures are devoid of information so they cannot encode thoughts. According to EMF-ToCs, they represent a kind of consciousness brain-wipe that is entirely consistent with the loss of consciousness in absence seizures.

7. Compared to neural-ToCs, EMF-ToCs have a distinct advantage in tackling the measurement problem, since, as outlined above, measurement of brain EMFs by EEG or MEG are routinely used to detect signs of consciousness in anesthesia (Pistoia et al., 2015; Schartner et al., 2015; Bayne et al., 2016; Hajat et al., 2017; Eagleman et al., 2018) and in disorders of consciousness, such as locked-in syndrome (Voss and Sleigh, 2007; Rohaut et al., 2017). Indeed, brain-computer interfaces (McFarland and Wolpaw, 2017; Nolte, 2021) that detect EEG signals have recently been developed to restore communication and control to people paralyzed by chronic neuromuscular disorders and allow locked-in patients to communicate *via* their (conscious) EEG signals. This is doubly-puzzling as, according to the Grand Illusion Hypothesis, our conscious mind only processes a tiny fraction of the information being processed by our non-conscious brain (Noë, 2002). No neural-ToC can account for why EEG and MEG signals are so well correlated with that thin conscious trickle rather than the bulk of non-conscious brain activity; but it is easily accounted for in EMF-ToCs that predict that these EMF measurement will correlate with the activity of the conscious mind. EMF_1_-ToCs predict that an EMF-encoded information loop is required for conscious control of actions. This is something that is potentially detectable by experiments that measure the degree by which external EM fields, of similar strength and structure but of opposite phase as brain/organoid/AI-generated EM fields, influence the outputs of neuronal, organoid or artificial computational devices by, essentially, neutralizing conscious inputs. The level of interference would then provide a measure of the degree of conscious control of behavior. Although experimentally challenging, experiments have demonstrated that external fields of similar strength and structure as endogenous EM fields do influence neural firing patterns in brain slices (Frohlich and McCormick, 2010; Anastassiou et al., 2011). Similar experiments performed on live animals would be able to test if external EM fields of similar strength and structure, but of opposite phase, as endogenous brain EM field are capable of influencing behavior. The degree of influence would be a measure of the level of conscious control of behavior. Since EMF_0_-ToCs do not predict that EM fields influence behavior, they cannot, as far as I am aware, be evaluated to determine level of awareness.
8. How the same neuronal architecture delivers both a massively-parallel non-conscious mode and a serial conscious mode of operation is not accounted for in any neuronal-ToC except through the imposition of arbitrary thresholds for conscious processing as being the most complex, integrated or those which involve particular anatomical sites or processing architecture, such as recurrent networks. Yet there is no evidence that computations routinely performed by the non-conscious mind, such as computing limb movements during walking or running, or orchestrating the delicate motions of tongue, lips and larynx required for speech or song, are any simpler, or less integrated, than those involved in conscious deliberations such as doing long division in one's head—a task that is easy for the simplest pocket calculator. Moreover, following the dialogue and action in a movie or theater surely requires a high degree of complex and highly integrated distributed neuronal processing of multiple sensory sources; yet it can easily be supplanted in the conscious mind by the simplest of stimuli, such as when a fellow audience member stands on your toe whilst shuffling past your seat. Global workspace theory (GWT) (Baars, 2005) and the related global neuronal workspace (GNWT) theories (Dehaene, 2014) avoid these pitfalls by not specifying the criteria by which neuronal activity gains access to the global workspace (Baars and Franklin, 2003) except, in GNWT, to claim that it is driven by non-linear feedback systems that flip between different states in a winner-takes-all dynamics (Dehaene, 2014). A related problem is to understand why activities, such as conversation or long division, can only be performed consciously (Dehaene, 2014).

EMF-ToCs provide a clearly defined physical distinction between matter-based EMF-independent non-conscious neural processing and EMF-dependent conscious information processing in the brain. Matter-based neuronal networks—the non-conscious mind—can easily partition tasks into spatially separated matter-based sub-networks that do not interfere with each other. The brain's EM field is however a singular entity so the conscious mind can only do one thing at a time. The second aspect of this criterion is to understand why conscious control is required to provide fine tuning in the process of learning, similar to what James (1988) envisaged more than a century ago (as quoted in [40]) suggesting that “if consciousness can load the dice, can exert a constant pressure in the right direction, can feel what nerve processes are leading to the goal, can reinforce and strengthen these and at the same time inhibit those that threaten to lead astray, why, consciousness will be of invaluable service”. In the cemi field theory, this is accomplished by the brain's EMF pushing and pulling on neurons toward or away from firing to achieve the desired motor actions. However, so long as target neurons are connected by Hebbian synapses then the repeated influence of the brain's EMF to accomplish a practiced action will tend to become hard-wired into either increased (long-term potentiation, LTP) or decreased (long-term depression) neural connectivity between the neurons involved in this action. After repeated augmentation by the brain's EMF, motor actions that were once painfully conscious may thereafter be performed non-consciously. The action is now learned and hardwired so no longer requires EMF input for fine-tuning. Type 1 EMF-ToCs thereby provide an entirely naturalistic account of why consciousness is intimately involved in learning and memory but, once learnt, is dispensable and may even interfere with performance.

9. EMF-ToCs distinguish between consciousness and intelligence, which is substrate independent and can be delivered by any matter or field-based information processing system, from neuronal networks to electronic circuits, intelligent materials or insect social networks (MacLennan, 1999); as well as in conscious and non-conscious minds. Non-classical states of matter, such as the Bose-Einstein condensates used in some quantum computers (MacLennan, 2022) do physically integrate matter-based information and could potentially integrate complex information in an analogous manner to brain EM fields. It remains to be seen whether there is something *it feels* to be a quantum computer.
10. The cemi field theory accounts for the emergence of consciousness through natural selection. Neurones in a complex brain display a range of excitability and in the busy brain of our ancestral animals there would have been many neurones poised close to their threshold potential with voltage-gated ion channels sensitive to small changes in the EM fields generating by surrounding neural activity. So long as they impacted neural firing—as has amply been demonstrated in studies referenced above—then those field interactions would have been subject to natural selection. Wherever field effects boosted performance, natural selection would have acted to enhance neurone sensitivity, for example, by maintaining neurones close to firing potential, decreasing nerve myelination or orientating and synchronizing neurones to maximize constructive interference. Potential advantages provided by EMF-based computing include, as outlined above, field computing, conscious fine-tunable learning and the ready availability of an EMF-based global workspace that can be accessed by the entire brain. In my previous publications, I have argued that the principle advantage captured by the conscious mind was to compute with the integrated packages of gestalt information that we call thoughts, rather than with the binary digits that are processed by the non-conscious mind and AI devices (McFadden, 2013b, 2020). Conversely, field influences were also likely to be detrimental to the host though EM field “feed-back” that interfered with informational processing of essential motor functions, such as reflex actions, together with learnt motor actions such as walking, running, or speech. It is easy to experience this kind of negative interference by attempting to exercise conscious control of our limb movements whilst engaged in a learnt and normally automatic motor task, such as walking or playing a musical instrument. For these EMF-impaired operations, natural selection would have acted to decrease EMF sensitivity by, for example, maintaining neurones far from firing potential, increasing myelination or orientating and desynchronising neurones to maximize destructive interference, as in the cerebellum. So, with only the information that endogenous EM fields influence neural firing, the theory of natural selection predicts that brains will evolve into an EM field-sensitive (conscious) system and a parallel EM field-insensitive (non-conscious) system. *Homo sapiens* have clearly followed this route. Note also that the region of the brain that is most involved in control of reflex actions, the cerebellum, is, as discussed above, also the most invisible to EEG because it produces only very weak EM fields that are unlikely to generate any EMF-feedback. As far as I am aware, it is only EMF_1_-ToCs that, in combination with the theory of natural selection, predict, entirely naturalistically and with no further assumptions, the inevitable evolutionary emergence of a parallel-computing non-conscious mind together with a serial computing conscious mind.
11. Whether the ToC is able to make novel testable predictions. I have already outlined the very strong prediction of EMF-ToCs that AIs based on conventional computing will never be conscious. Moreover, because EMF's are subject to wave interference, EMF-ToCs make another strong prediction that changing only the relative timing of neuronal firings will affect conscious perception. For example, it should be possible to switch between the alternative conscious perceptions of the face/vase illusion merely by shifting the relative timings of neuronal signals involved in generating the brain EM fields that correlate with the alternative perceptions through constructive or destructive interference. In this way, the brain could be manipulated so that information encoded in neuron firing rates is always present in the neuronal brain but alternatively present/absent in brain EM fields. EMF-ToCs predict that consciousness will correlate with the informational content of the brain's EM field, rather than its neurons, whereas all neural-ToC's predict the opposite. Recent advances in application of optogenetic techniques (Toettcher et al., 2011; Boyden, 2015; Adesnik and Abdeladim, 2021) in humans and non-human primates (Han, 2012) are likely to make such an experiment a real possibility in the near future.

Other prediction of EMF-ToCs have already been verified, albeit unintentionally. For example, as described above, the cemi field theory proposes that EM fields are involved in memory and learning and so would predict that external EM fields, such as those delivered by TMS, will interfere with these processes, as found in several studies (Ferrari et al., 2018; Bang et al., 2019) but will be impervious to external fields once learnt, as has also been demonstrated (Bang et al., 2019). Even more remarkably, a recent study has demonstrated that retrieval of human memories involved coupled ripple oscillations in the EEG between the medial temporal lobe and the neocortex (Vaz et al., 2019).

## Discussion

At first sight, EMF-ToCs appear far-fetched: how can EM fields be conscious? But is it any more unreasonable to propose that the matter of the brain is conscious? In Terry Bison's delightful short story “They're Made out of Meat” (see opening quotation), two aliens ponder the shocking discovery of a “meat”-based sentient species inhabiting a planet in a remote corner of the galaxy (Bisson, 1995). One of the aliens' proposes that there must be more to the new species, perhaps “A meat head with an electron plasma brain inside” but the other insists that “they're meat all the way though... Yes, thinking meat! Conscious meat! Loving meat. Dreaming meat. The meat is the whole deal!” They agree to suppress the new data.

Most neurobiologists continue to believe that the matter of the brain, its flesh, is the whole deal despite knowing for more than a century, that, alongside the particles of matter that make up the brain's visible matter, there is also the equally physical, though invisible, electromagnetic field generated by neuronal firing, action potentials and synaptic transmission. Modern particle physics tells us that those particles—protons, electrons, neutrons—that make up the matter of the brain—are actually excitations of underlying electromagnetic, weak and the strong nuclear force fields, together with the Higgs field. Moreover, apart from physical processes involving radioactive decay or gravity, pretty much everything that happens on our planet, all of chemistry and the biochemistry of life, is mediated by electromagnetic field interactions. Is it really so bizarre to propose that some of those interactions are also the substrate of life's greatest gift, consciousness?

Of course, it remains to be proved that the brain's electromagnetic field is the substrate of consciousness. But then it also remains to be proved that the matter of the brain is the substrate of consciousness. As far as I am aware, there is no experiment that favors the brain's matter, as the substrate of consciousness, over its EM fields. Yet, as outlined above, EMF-ToCs provide the most parsimonious accounts of numerous phenomenal aspects of consciousness, including its serial nature, binding and the disconnect between intelligence and consciousness. EMF-ToC's explain why consciousness is involved in learning together with an account of the evolution of consciousness which predicts one of the mind's most curious features, that it operates in both non-conscious (parallel) and conscious (serial) modes. EMF-ToCs achieve all this without recourse to any special states of matter, hypothetical workspaces, or impenetrable equations. It is surely time for neurobiologists to accept that there is more to mind than matter.

## Data availability statement

The original contributions presented in the study are included in the article/supplementary material, further inquiries can be directed to the corresponding author.

## Author contributions

The author confirms being the sole contributor of this work and has approved it for publication.

## Footnotes

^1^The subscripted numbers represents the binary possibilities of influencing (1) or not influencing (0) behaviour.
